# Magnetically Driven Manipulation of Nonmagnetic Liquid Marbles: Billiards with Liquid Marbles

**DOI:** 10.3390/mi14010049

**Published:** 2022-12-25

**Authors:** Parnian Azizian, Mahbod Mohammadrashidi, Ali Abbas Azimi, Mohamad Ali Bijarchi, Mohammad Behshad Shafii, Rohollah Nasiri

**Affiliations:** 1 Department of Mechanical Engineering, Sharif University of Technology, Tehran 11155-9567, Iran; 2 Department of Protein Science, Division of Nanobiotechnology, KTH Royal Institute of Technology, 171 65 Solna, Sweden

**Keywords:** ferrofluid, liquid marble, digital microfluidics, magnetic manipulation, liquid marble manipulation

## Abstract

Liquid marbles are droplets encapsulated by a layer of hydrophobic nanoparticles and have been extensively employed in digital microfluidics and lab-on-a-chip systems in recent years. In this study, magnetic liquid marbles were used to manipulate nonmagnetic liquid marbles. To achieve this purpose, a ferrofluid liquid marble (FLM) was employed and attracted toward an electromagnet, resulting in an impulse to a water liquid marble (WLM) on its way to the electromagnet. It was observed that the manipulation of the WLM by the FLM was similar to the collision of billiard balls except that the liquid marbles exhibited an inelastic collision. Taking the FLM as the projectile ball and the WLM as the other target balls, one can adjust the displacement and direction of the WLM precisely, similar to an expert billiard player. Firstly, the WLM displacement can be adjusted by altering the liquid marble volumes, the initial distances from the electromagnet, and the coil current. Secondly, the WLM direction can be adjusted by changing the position of the WLM relative to the connecting line between the FLM center and the electromagnet. Results show that when the FLM or WLM volume increases by five times, the WLM shooting distance approximately increases by 200% and decreases by 75%, respectively.

## 1. Introduction

In recent years, liquid marbles have developed a reputation as an alternative to conventional naked droplets in digital microfluidic systems [1]. Liquid marbles are droplets encapsulated by layers of hydrophobic micro/nanoparticles and were first introduced in the revolutionary work of Aussillous and Quere [2,3,4]. When a liquid droplet rolls on a hydrophobic powder bed, due to the tendency of the system to minimize its overall energy level, these particles adhere to the droplet interface, and thus a liquid marble forms. As can be inferred from their name, liquid marbles act as a semi-solid and semi-liquid, and due to the presence of a particle shell around the liquid core, liquid marbles are less prone to sample contamination, have a slower evaporation rate, and do not need a hydrophobic surface to be manipulated as compared to simple droplets [1]. All these advantages have made liquid marbles an excellent choice as microbioreactors for chemical or biological assays [5,6,7]. 

In the literature, several methods have been proposed for manipulating liquid marbles [1,8,9,10,11]. Gravitational force has been widely used for the actuation of liquid marbles [2,3]. Wang et al. have developed an intricate, three-dimensional, and gravity-based device to coalesce and split liquid marbles [12]. Moreover, some electrical schemes have matured to manipulate liquid marbles [13]. Ooi et al. have investigated the capability of exploiting dielectrophoresis to manipulate floating liquid marbles [14]. Similarly, an electrowetting on a dielectric (EWOD) platform could be used for liquid marble actuation [15,16]. In addition to the aforementioned schemes, acoustic waves [17,18], optical waves [19], and magnetic force [20] are among the most renowned methods for the control and actuation of liquid marbles. 

However, magnetic actuations have certain advantages over the mentioned schemes. For instance, magnetic actuation methods are simple, wireless, nonintrusive, inexpensive, and not dependent on the pH and ions of the sample. As a result, the exploitation of an external magnetic field is emerging as a versatile method for the manipulation of liquid marbles [20]. In order for liquid marbles to be responsive to a magnetic field, hydrophobic magnetic particles can be used as the outer shell of liquid marbles [21,22,23]. Zhao et al. studied the use of hydrophobic magnetite nanoparticles for the formation of magnetic liquid marbles. They enwrapped water droplets with hydrophobic Fe_3_O_4_ nanoparticles and showed that such liquid marbles could be manipulated with a permanent magnet [21]. Interestingly, they observed that the magnetic shells of these liquid marbles open reversibly if a magnet approaches the bottom of the liquid marbles. This reversible opening and closing of the liquid marble shells has applications in the optical probing of the liquid core of liquid marble [23]. In addition to the outer shell of liquid marbles, magnetic materials can be implemented in liquid marble cores to form magnetic liquid marbles. Khaw et al. used a mixture of deionized water and magnetite powder as the core of liquid marbles and investigated the dynamic behavior of the resulting liquid marbles floating on a water surface [24,25]. Not only water but also liquid metals can be used as liquid marble cores. Jeong et al. capsulated liquid metal droplets with magnetic particles and studied the manipulation of these liquid metal marbles [26].

Ferrofluid, a stabilized suspension of magnetic nanoparticles in a fluidic media [27,28], has been found to have a myriad of applications in microfluidic systems thanks to its controllability by an external magnetic field [20,29,30,31,32,33,34,35,36,37,38,39,40,41,42]. In the last decades, researchers have comprehensively studied the manipulation of ferrofluid droplets [43,44,45,46,47,48,49,50]. Nguyen et al. utilized an array of microcoils to manipulate ferrofluid droplets [46]. Nguyen et al. also studied the magnetowetting phenomena and sliding motion of ferrofluid sessile droplets [47]. Katsikis et al. studied the logical manipulation and control of ferrofluid droplets immersed in an oil-based medium under a rotating magnetic field [48]. Bijarchi et al. exploited an alternating magnetic field to manipulate ferrofluid droplets and studied the motion of these droplets under a pulse-width modulated magnetic field [49]. Latikka et al. introduced a method for the population-based splitting of ferrofluid droplets with applications in measuring ferrofluid surface tension [50]. 

Ferrofluid droplets themselves can be utilized as manipulators for the transportation and actuation of other nonmagnetic objects, droplets, or samples. In a novel study conducted by Fan et al., ferrofluid droplets were introduced as multifunctional robots capable of manipulating and carrying delicate objects [51]. Mandal et al. showed the potential of oil-based ferrofluid droplets to transport an aqueous sessile droplet mounted on the ferrofluid droplet spikes [52]. In a recent study, Lin et al. developed a ferrobotic framework in which ferrofluid droplets were utilized as an intermediate fluid for sample transportation, aliquoting, merging, mixing, and heating in SARS-CoV2 virus clinical detection tests [53]. 

Ferrofluid droplets could be enwrapped by hydrophobic micro/nanoparticles to construct ferrofluid liquid marbles. A ferrofluid liquid marble has the advantages of maneuverability and controllability under external magnetic stimuli as well as other intrinsic advantages of liquid marbles. Bormashenko et al. covered a ferrofluid droplet with polyvinylidene fluoride nanoparticles and showed the controllability of the resulting liquid marble with a permanent magnet [54]. Nguyen studied the static and dynamic deformation of ferrofluid liquid marbles under a magnetic field generated by a permanent magnet [55]. Despite these outstanding studies, there is still much room for investigating the potential capabilities and applications of ferrofluid liquid marbles in digital microfluidic systems. 

To the best of our knowledge, ferrofluid liquid marbles (FLMs) have not been previously used as actuators for the manipulation of other objects. In fact, the FLMs utilized in this study act as a robot capable of manipulating other objects, particularly nonmagnetic liquid marbles. Moreover, one of the objectives of this study is to introduce a manipulation scheme for utilization in biomedical platforms, such as polymerase chain reactions (PCRs); however, FLMs have biocompatibility limitations that prevent them from being directly utilized for biomedical applications [56]. Therefore, in this study, for the first time, a magnetoresponsive ferrofluid liquid marble was utilized to manipulate a nonmagnetic water liquid marble (WLM). This study used an adjustable electromagnet to attract an FLM toward a WLM, and the collision of the FLMs and WLMs was comprehensively investigated. As a result of the impact, the driving FLM could shoot the driven WLM to various distances and directions, analogous to balls in a billiards game, and the effect of different independent parameters, including the liquid marbles’ volumes, their initial distances from the electromagnet, and the coil current, on the impact was experimentally examined. Our proposed method for WLM shooting has potential applications in digital microfluidic systems for large-distance sample transportation or for taking samples out of chips.

## 2. Materials and Methods

**Figure 1:**
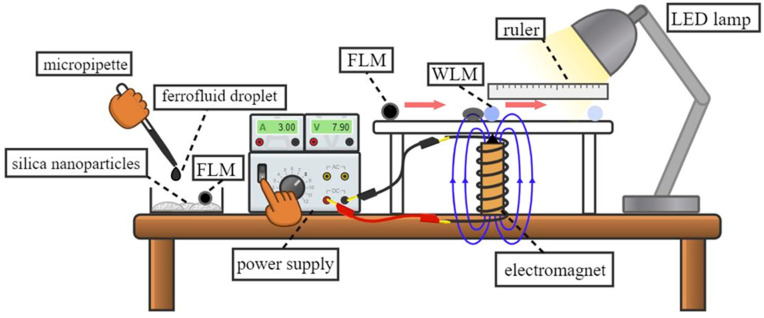
Schematic of the apparatus.

Figure 1 depicts the schematic of the apparatus. In this study, water-based ferrofluid MSG-W10 (Ferrotec, Santa Clara, CA, USA) was employed to form the FLMs. The magnetic particles dispersed in the ferrofluid had a nominal diameter of 10 nm and a volumetric concentration of 3.6%. The nominal ferrofluid physical properties, including density, dynamic viscosity, and surface tension, were reported as 1190 kg/$m^{3}$, 2.8 mPa·s, and 40 mN/m, respectively. Moreover, initial magnetic susceptibility and saturated magnetization were measured to be 0.67 and 14,722 A/m, respectively. Additionally, for the WLM formation, deionized water with density, dynamic viscosity, and surface tension of 998 kg/$m^{3}$, 0.9 mPa·s, and 72 mN/m was utilized.

In this study, liquid marbles with volumes ranging from 10 μL to 50 μL were utilized. This range of volume ensured that both spherical (Bo_g_ < 1) and puddle-shaped (Bo_g_ > 1) liquid marbles were inspected. The formation of WLMs and FLMs consisted of dispensing the water or ferrofluid droplets with a micropipette on a powder bed of hydrophobic silica nanoparticles with 20–30 nm in diameter and then shaking the powder container so that the droplets completely rolled on silica nanoparticles, as can be observed in the left side of Figure 1. This method ensures that the silica nanoparticles adhered to the outer surface of the droplets, and thus the liquid marbles were perfectly established. Afterward, WLMs and FLMs were transferred onto a glass with a thickness of 2 mm, which was placed on top of a 1 mm thick PMMA stand. The height of the PMMA stand was regulated so that the lower surface of it came into contact with the top of the electromagnet. Therefore, a vertical distance of 3 mm between the upper surface of the glass and the top of the electromagnet was attained. Moreover, with the aid of the lines and grids on a grid paper stuck beneath the glass surface, the positions of FLM and WLM relative to the coil tip could be manually adjusted with a maximum error of ±0.5 mm.

**Figure 2:**
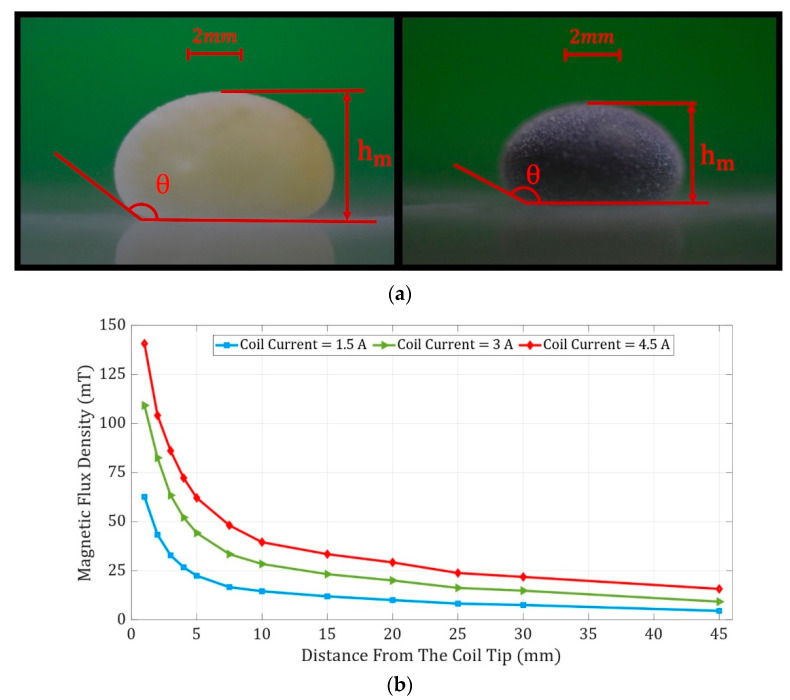
(**a**) Sideview of the WLM and FLM with volumes of 150 μL and 250 μL, respectively. (**b**) The magnetic flux density versus the horizontal distance from the coil tip for coil currents of 1.5 A, 3.0 A, and 4.5 A. The vertical distance between the top of the coil and the plane on which FLMs were placed is fixed at 3 mm.

The maximum puddle height method was utilized to obtain the effective surface tension of the liquid marbles [57]. In this approach, several sideview images from liquid marbles with various volumes were captured. When FLMs exceeded 150 μL in volume, their height did not increase by more than 0.5%. As a result, the maximum height and contact angle of FLMs were measured to be 3.71 mm and 152 degrees, respectively (Figure 2a). By employing Equation (1), the effective surface tension of FLMs was measured to be 42.2 mN/m. Furthermore, the height of WLMs did not change remarkably when their volumes exceeded 250 μL. Therefore, the WLMs maximum height and contact angle were observed to be equal to 5.15 mm and 145 degrees, respectively. Again, based on Equation (1), the effective WLM surface tension was 71.3 mN/m. In this equation, σ, ρ, g, h_m_, and θ are effective surface tension (N/m), liquid marble density (kg/$m^{3}$), earth gravitational acceleration (m/$s^{2}$), the maximum height of the liquid marbles (m), and contact angle (degree), respectively.

(1) $$\sigma =\frac{{\rho \mathrm{gh}}_{m}^{2}}{2\left(1-\cos\theta \right)}$$

The electromagnet mentioned previously consisted of a copper wire with a 1 mm diameter wound around a ferrite core. A magnetic field was generated as an electric current generated by a MICRO, PW-4053S power supply passing through the coil wire. Since magnetic field lines were concentrated at the top of the pyramid-shaped ferrite core, the manipulation and actuation of FLMs were precisely controlled. A gaussmeter (MG 3002-LUTRON) with 0.01 mT accuracy was utilized to measure the magnetic flux density for different vertical distances from the coil tip and various currents passing through the coil, as shown in Figure 2b.

Liquid marble motion was recorded with a CCD camera (Nikon1 J4) with a resolution of 288 × 768 and a frame rate of 400 fps. The length-to-pixel ratio was measured to be 72 μm/pixel in this work. Moreover, the image processing software developed by Basu was utilized for processing recorded images [58]. 

This study investigated the manipulation of a WLM by an FLM. To achieve this objective, the FLM played the role of a driver liquid marble, the WLM was regarded as a driven liquid marble, and the impulse and collision of the two liquid marbles were precisely studied. In order to force the FLM to move toward the WLM and collide with it, an electromagnet was employed to attract the FLM, and the WLM was placed in the way of the FLM as it approached the electromagnet (Figure 1). 

Therefore, since the WLM was placed on the connecting line between the FLM center and the electromagnet, it would be launched as the FLM moved toward the electromagnet, exhibiting a motion similar to billiard balls. More specifically, the FLM could be a substitute for the projectile ball, and the WLM could be related to other target balls in a billiards game that is launched after the impulse of the projectile ball (FLM). The current study investigated this quasi-billiard shooting motion, particularly the distance and the direction that the WLM launched after the impulse of the FLM.

**Figure 3:**
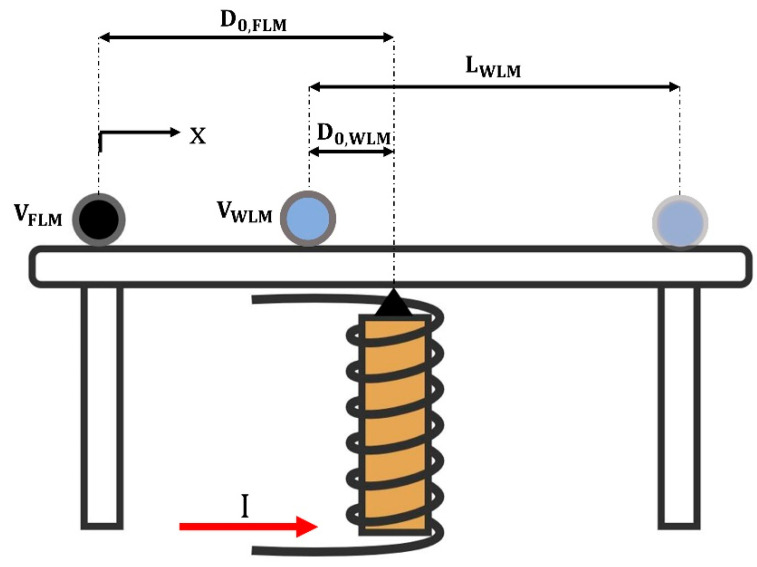
Schematic of the WLM shooting distance ($L_{\mathrm{WLM}})$ in addition to five parameters in this study, including the initial distances of WLM and FLM from the electromagnet ($D_{0,\mathrm{wlm}\;}$,$D_{0,\mathrm{FLM}})$, the WLM and FLM volumes ($V_{\mathrm{WLM}}$, $V_{\mathrm{FLM}})$, and the coil current (I).

The WLM shooting distance ($L_{\mathrm{WLM}})$ after collision with the FLM depends on many parameters, some of which are the initial distances of WLM and FLM from the electromagnet ($D_{0,\mathrm{wlm}\;}$,$D_{0,\mathrm{FLM}})$, the WLM and FLM volumes ($V_{\mathrm{WLM}}$, $V_{\mathrm{FLM}})$, and the coil current (I). In this study, the change in WLM shooting distance was investigated by altering these five parameters, as shown in Figure 3, and the physical interpretation of the obtained results is then discussed. 

The initial position of WLM was fixed at specific distances from the electromagnet, starting from the top of the electromagnet to 2 cm away from it. Likewise, the FLM initial distance from the electromagnet decreased from 5 cm to 1 cm. Moreover, the WLM or FLM volume could be adjusted by changing the volume of water or ferrofluid droplets from 10 μL to 50 μL using a micropipette. The last parameter, magnetic flux density, could change by altering the current passing through the electromagnet wire from 1 A to 5 A. 

## 3. Results and Discussion

**Figure 4:**
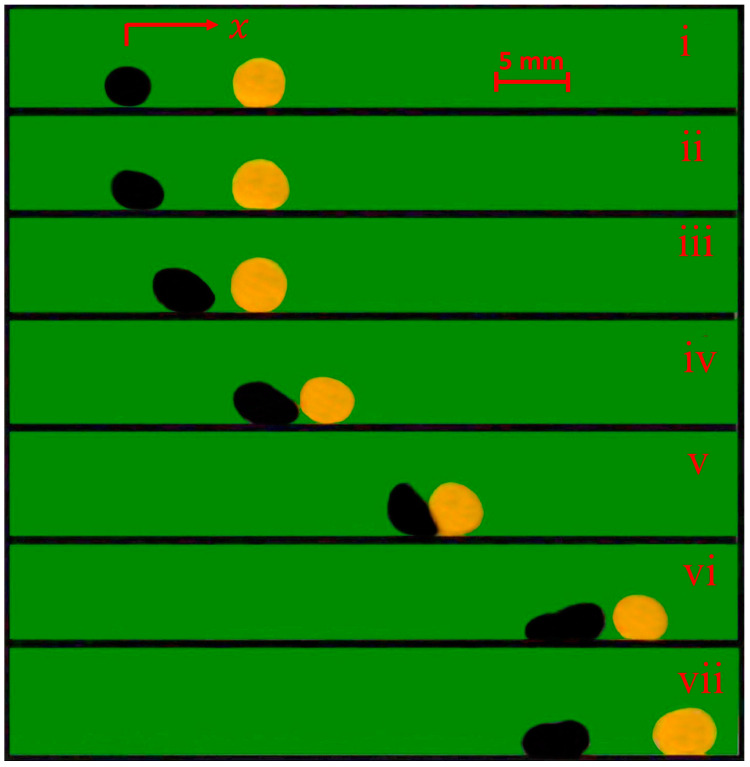
The process of launching a WLM with an FLM with volumes of 30 μL, initial distances from the electromagnet of 10 mm and 30 mm, respectively, and the coil current of 3 A. (i–iii) FLM moves toward the electromagnet while WLM is stationary. (iv, v) FLM impacts the WLM, and LMs stick and move together toward the electromagnet. (vi–vii) FLM stops at the top of the coil, while WLM continues its motion due to its high inertia.

The manipulation of the WLM by the FLM consisted of four parts. Firstly, the FLM moved toward the electromagnet with a positive acceleration while the WLM was at rest. Afterward, during the inelastic collision in which the two objects almost stuck and moved together after impact and a portion of their kinetic energy was dissipated [59], the driver FLM impacted the driven WLM and transferred a portion of its momentum to the WLM. Then, the stuck-together liquid marbles moved toward the electromagnet and acquired momentum in the meantime. This motion continued until the two liquid marbles reached the top of the electromagnet. Afterward, the FLM crossed the coil tip due to its high inertia, but it is attracted back toward the coil due to the continuously applied magnetic force. Eventually, the FLM stopped at the top of the coil after a few oscillations around the coil tip. In contrast, the WLM continued to move even after reaching the electromagnet. Since the WLM is a nonmagnetic liquid marble, it was not attracted by the electromagnet and continued its motion due to its high inertia and low friction with the surface. This resulted in the WLM being released on the top of the electromagnet. Figure 4 and Video S1 illustrate the process of launching the WLM, including the motion of the FLM before the inelastic collision, the stuck-together liquid marbles’ movement toward the electromagnet, and the motion of the WLM after release. Although the two liquid marbles were not rigid bodies and were soft matter with the capability of being deformed (as can be observed in Figure 4 and Video S1), their collision could occur in an inelastic manner.

**Figure 5:**
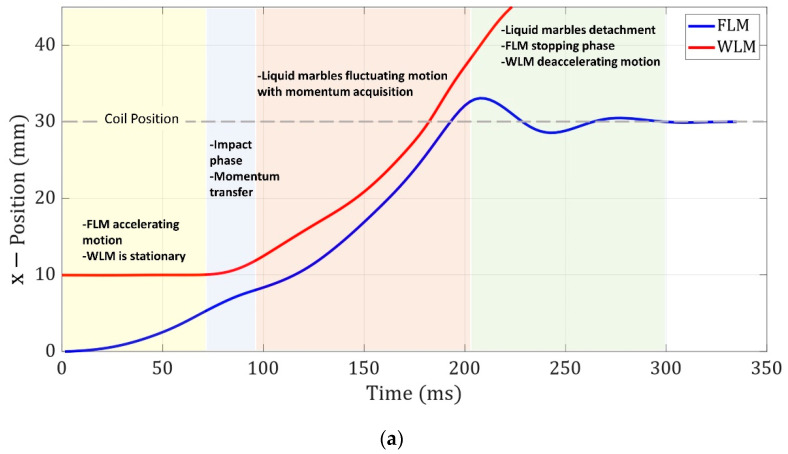

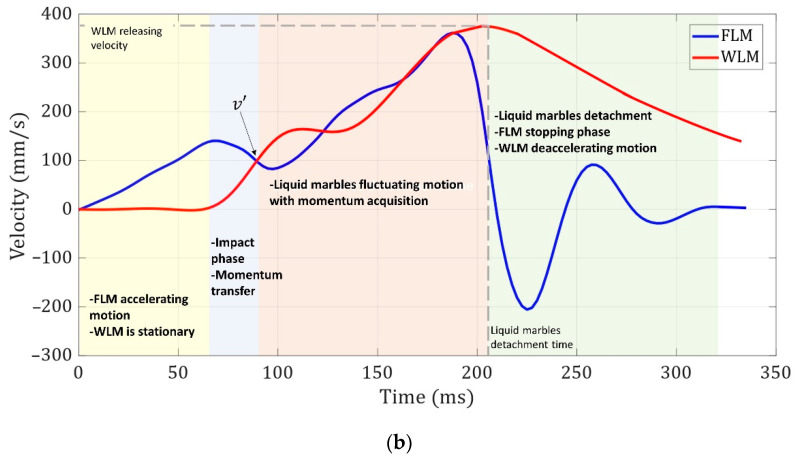
(**a**) Position and (**b**) velocity of FLM and WLM versus time for liquid marble volumes of 30 μL, coil current of 3 A, and FLM and WLM initial distances of 30 mm and 10 mm from the electromagnet, respectively.

In order to investigate the dynamics of two liquid marbles more precisely, the FLM and WLM positions and velocities before, during, and after the collision were reported. Figure 5a,b demonstrates the positions and velocities of the two liquid marbles in the four aforementioned stages, respectively. As can be observed in Figure 5b, the FLM exhibited a positive accelerating motion when it moved toward the electromagnet, leading to an increment in its velocity as it got closer to the electromagnet while the WLM was at rest. Afterward, the FLM impacted the WLM, and the two liquid marbles stuck together with a fluctuating deformation. Then, the system started moving toward the electromagnet with the same velocity ($v^{\prime })$, which was less than the FLM velocity right before the collision ($v_{1}$). The conservation law of linear momentum for this collision is derived in Equation (2). In this equation, $m_{f}$ and $m_{w}$ are the masses of the FLM and WLM. Furthermore, $v_{1}$ and $v^{\prime }$ are the FLM velocity right before the collision and the FLM and WLM velocity right after the collision, respectively.

(2) $$m_{f}v_{1}=\left(m_{f}+m_{w}\right)v^{\prime }\to \frac{v^{\prime }}{v_{1}}=\frac{m_{f}}{m_{f}+m_{w}}<1$$

Nonetheless, the velocity v’ increased over time because of the system momentum acquisition. In addition, the two liquid marbles’ velocities fluctuated relative to each other due to their deformable behavior. Afterward, when the two liquid marbles reached the electromagnet, the FLM stopped moving and became stationary. On the other hand, the WLM continued to move since it was not affected by the magnetic field. After passing the electromagnet and being released from the FLM, the WLM showed a deaccelerating motion due to the friction force against its motion and finally stopped moving and attained zero velocity.

**Figure 6:**
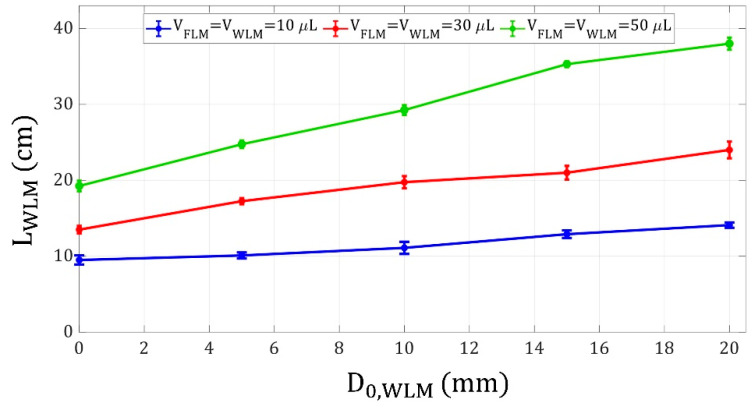
WLM shooting distance versus WLM initial distance from the electromagnet for liquid marble volumes of 10, 30, and 50 μL, FLM initial distance of 30 mm from the electromagnet, and coil current of 3 A.

Herein, the WLM shooting distance was investigated by altering the five parameters mentioned in the Materials and Methods section. Firstly, the effect of changing the WLM initial position on the WLM shooting distance was studied. Figure 6 demonstrates the variation of the WLM shooting distance versus the WLM initial distance from the electromagnet for different liquid marble volumes. 

It is worth considering the linear impulse–momentum principle in order to understand this effect (Equation (3)). The moment exactly before turning the electromagnet on, when the FLM and WLM are at rest, is regarded as *t* = 0. The moment when the stuck-together liquid marbles reach the electromagnet can be considered as time *t* = T. According to Equation (3), the magnetic force applied to the system consisting of two liquid marbles leads to an increment in the linear momentum of the system from *t* = 0 to *t* = T. In this equation, $v_{w,1}$, $v_{f,1}$, $v_{w,2}$, and $v_{f,2}$ are the velocities of the WLM and FLM at the times *t* = 0 and *t* = T, respectively. Furthermore, F is the net force applied to the FLM while moving toward the electromagnet. In general, F represents all the forces applied to the system, such as the magnetic driving force, forces due to the viscous dissipations and triple line hysteresis, and the air drag force. However, due to the low-friction nature of liquid marbles [1,2,3] and for the simplicity of the proposed model, all frictional forces are neglected, and thus F only represents the applied magnetic force. It should be mentioned that as the liquid marbles move on the surface, a trail of particles remains on the surface. Moreover, some particles are ejected from the liquid marbles as they collide with each other. Nonetheless, due to the negligible mass of these exhausted particles, the mass of liquid marbles is assumed to be constant.

(3) $$m_{w}v_{w,1}+m_{f}v_{f,1}+\int_{0}^{T}\mathrm{Fdt}=m_{w}v_{w,2}+m_{f}v_{f,2}$$

Since the two liquid marbles are initially at rest ($v_{w,1}={\;v}_{f,1}=0$) and the collision of WLM and FLM is considered an inelastic collision with the same final velocity ($v_{w,2}={\;v}_{f,2}={\;v}_{2}$), the linear impulse–momentum principle can be simplified to Equation (4):

(4) $$\int_{0}^{T}\mathrm{Fdt}=(m_{w}+m_{f})v_{2}$$

From this equation, it can be concluded that the more time it takes the liquid marbles to reach the electromagnet from the moment the FLM starts moving, the more their final velocity will be, and thus the more the WLM will be launched. From a physical point of view, if the system of liquid marbles has more time to move together, the system acquires more momentum from the external force applied to the system. Therefore, the WLM will have more momentum in the final stage, leading to a greater WLM shooting distance.

Similarly, suppose the WLM initial position is farther from the electromagnet; thus, it is closer to the FLM with a fixed distance from the electromagnet. In that case, the FLM will collide with the WLM in a relatively short amount of time. Still, it will take the liquid marbles a longer time interval to reach the electromagnet since the WLM acts as an external load, preventing the FLM from moving freely toward the electromagnet. In this case, the time interval between 0 and T will be greater, resulting in a higher final velocity of the system including both the WLM and FLM. Consequently, since the WLM releasing velocity on the top of the electromagnet is higher, it can move farther until it fully stops due to the existence of friction force, leading to a greater WLM shooting distance. 

Moreover, in Figure 6, it is observed that the WLM shooting distance increases with an increment in WLM and FLM volumes. Two conflicting factors are affecting this phenomenon. Firstly, by increasing the WLM volume, its motion and launch might be harder due to its larger inertia and higher tendency to remain stationary. Secondly, by increasing the FLM volume, the magnetic force applied to it increases, leading to a greater WLM shooting distance. According to the results in Figure 6, the latter factor is dominant. Thus, an increment in the liquid marble volumes led to an increase in the WLM shooting distance.

**Figure 7:**
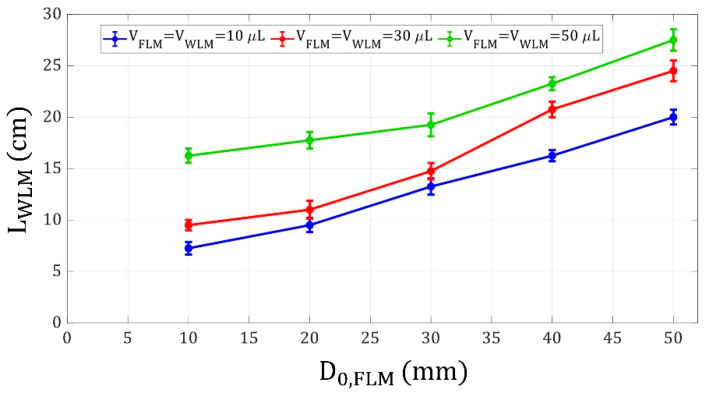
WLM shooting distance versus FLM initial distance from the electromagnet for liquid marble volumes of 10, 30, and 50 μL, WLM initial distance of 0 mm from the electromagnet, and coil current of 3 A.

Another parameter that significantly affected the motion of the driven liquid marble was the initial position of the FLM. Figure 7 shows the WLM shooting distance versus FLM initial distance from the electromagnet.

To investigate this effect, the velocity of the driver liquid marble when it impulses the driven one on the top of the electromagnet is critical. One can assume two cases such that in case A, the FLM is initially farther from the electromagnet than in case B. Due to the accelerating motion of FLMs when they are attracted to the electromagnet, the FLM in case A possesses a nonzero velocity when it reaches the FLM position in case B while the FLM in case B has an initial velocity of zero at that point. Moreover, because a liquid marble increases in velocity up to the top of the electromagnet, the liquid marble in case A attains a higher velocity when it reaches the electromagnet than the liquid marble in case B. Therefore, the FLM in the first case collides with the WLM above the electromagnet more strongly, resulting in a greater WLM shooting distance.

Furthermore, as discussed before, the effect of FLM volume is more significant than WLM volume. As shown in Figure 7, the larger FLMs launched the larger WLMs more strongly due to the larger magnetic force applied to them even though the WLMs with higher volumes could potentially cause a limitation in launching due to their larger inertia and lower acceleration compared to smaller WLMs. 

**Figure 8:**
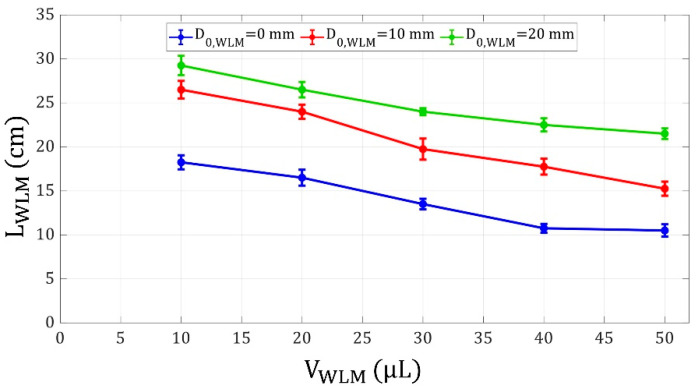
WLM shooting distance versus WLM volume for WLM initial distances of 0, 10, and 20 mm from the electromagnet, FLM volume and initial distance of 30 μL and 30 mm from the electromagnet, respectively, and the coil current of 3 A.

Moreover, the influence of WLM volume on its shooting distance was examined. With a constant force applied to a WLM by a specific FLM, the larger the WLM is, the less it is displaced as a result of that applied force. In other words, according to Newton’s law of motion, a larger WLM tends to maintain its stationary state more than a smaller one, and it is harder to accelerate a larger WLM with a constant force. Figure 8 also confirms that with an increment in WLM volume, its shooting distance decreases. In addition, as discussed before, the system of the FLM and WLM is disposed to the magnetic field for a longer interval if the WLM is placed closer to the FLM, and therefore, the system acquires more momentum from the magnetic field. Thus, the WLM has a higher velocity when it reaches the electromagnet, leading to a stronger launch and a greater shooting distance.

**Figure 9:**
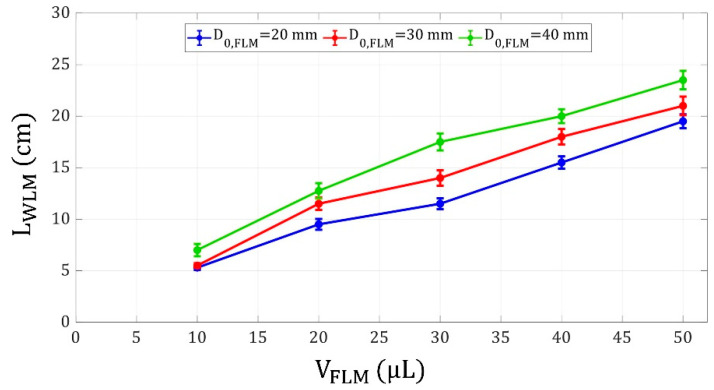
WLM shooting distance versus FLM volume for FLM initial distances of 20, 30, and 40 mm from the electromagnet, WLM volume and initial distance of 30 μL and 0 mm from the electromagnet, respectively, and coil current of 3 A.

Additionally, the effect of FLM volume on WLM shooting distance was studied. According to Figure 9, the WLM shooting distance increases by increasing the FLM volume. This is because as the FLM gets larger in size and mass, it impulses the WLM more strongly, resulting in a farther final position of the WLM. Furthermore, it was observed that with an increment in the FLM distance from the electromagnet, the WLM shooting distance increased as well. As mentioned before, Figure 9 also shows that at a constant FLM volume, by increasing the FLM initial distance from the electromagnet, the WLM shooting distance increases.

The last parameter investigated in this study was the coil current. The influence of this parameter on WLM motion can be related to the dependence of the magnetic flux density on the coil current. The magnetic flux density increases with an increase in the coil current, and according to Equation (5), a larger magnetic force to the FLM is applied [35,36].

(5) F=VM·∇B 

**Figure 10:**
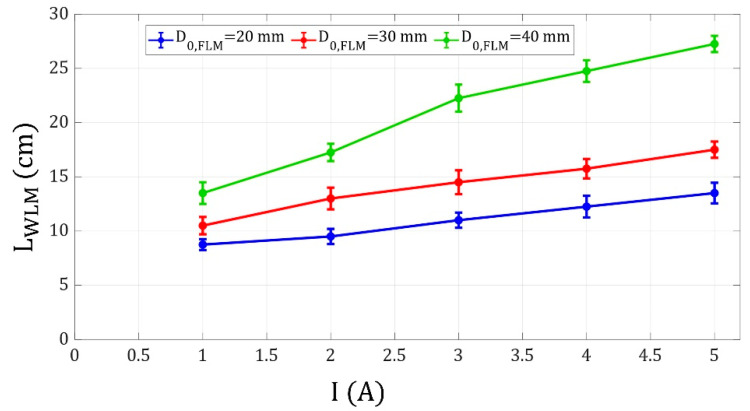
WLM shooting distance versus coil current for FLM initial distances of 20, 30, and 40 mm, WLM initial distance of 0 mm from the electromagnet, and liquid marble volumes of 30 μL.

In this equation, M is the ferrofluid magnetization, V is the FLM volume, B is the magnetic flux density generated by the electromagnet, and F is the magnetic body force applied to the FLM. According to Figure 10, as the coil current increases, the FLM is attracted toward the electromagnet with a larger force, leading to a stronger impact on the WLM on top of the electromagnet and a greater WLM shooting distance. Furthermore, the impulse of an FLM with a farther initial distance from the electromagnet is stronger than one with a closer initial distance based on the aforementioned elaboration. Therefore, by increasing in the FLM initial distance from the electromagnet (where the WLM is placed), the WLM shooting distance increases due to its stronger collision with farther FLMs.

**Figure 11:**
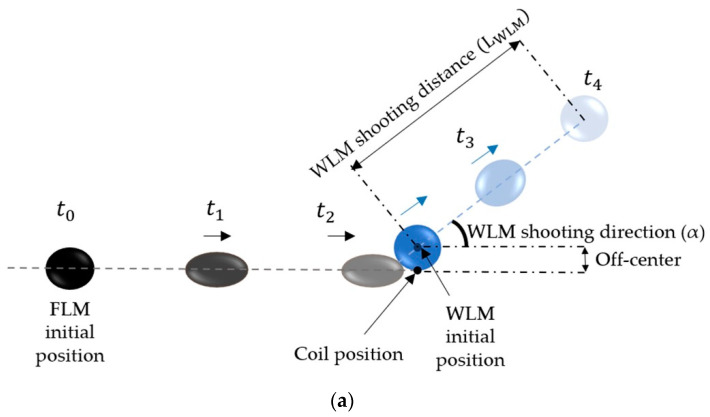

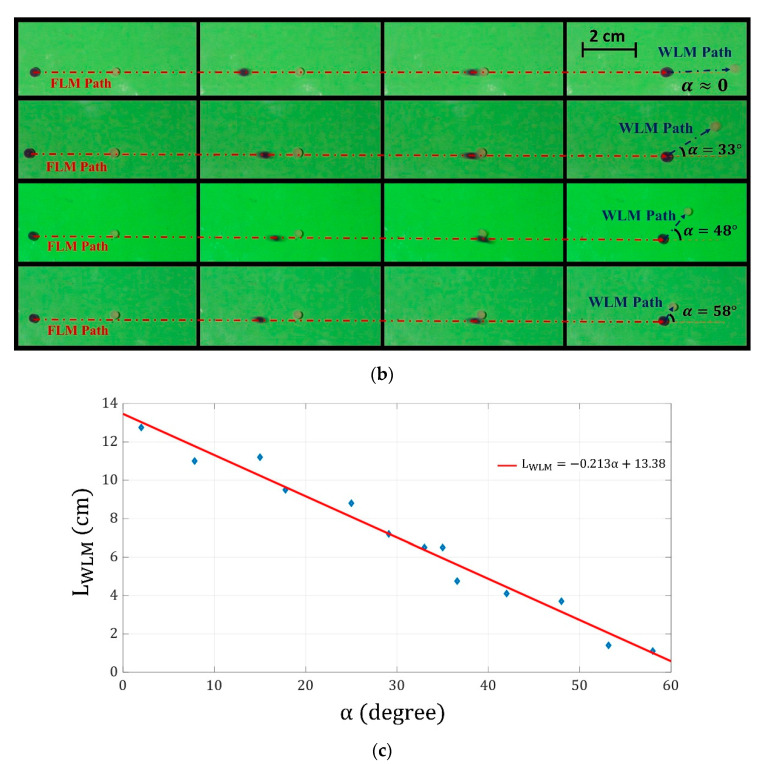
(**a**) Schematic of a WLM placed with an off-center position from the connecting line between the FLM center and the electromagnet tip, leading to a nonstraight WLM motion path. (**b**) A sequence of pictures of the straight and nonstraight collisions between FLM and WLM with volumes of 20 μL, initial distances of 30 mm and 0 mm from the electromagnet, respectively, and coil current of 3 A. (**c**) WLM shooting distance versus WLM shooting path degree from the FLM path (the red line is fitted linearly to the experimental data.).

At last, the two-dimensional manipulation of WLMs was investigated. In order to launch a WLM in different directions with different angles from the straight line in which the FLM moves, the center of the WLM can be placed off-center from the connecting line between the FLM center and the electromagnet tip, which leads to a nonstraight shooting of the WLM, as illustrated in Figure 11a. Particularly, the direction of the WLM’s movement can be adjusted by changing its off-center placement, enabling us to have thorough and precise control over the manipulation of the WLM. Similarly, a billiard player can adjust the direction of impact to the projectile ball in a way that the target ball moves in a favorable direction. 

Figure 11b demonstrates a series of pictures during the collision of liquid marbles with different off-centers. Accordingly, if the center of WLM is exactly on the connected line between the FLM center and the electromagnet tip (the FLM path), the WLM will be launched straightly. In contrast, if the WLM has an off-center, it will be launched no longer in the FLM path and will move with an angle (Video S2). 

In addition to the WLM direction, its shooting distance can be adjusted by changing the WLM off-center as well. According to Figure 11c, the more the off-center is, the less momentum is transferred from the FLM to the WLM, and the less WLM is launched.

## 4. Conclusions

This study investigates the manipulation of a WLM by an FLM under different conditions. Firstly, the effect of the WLM and FLM initial distances from the electromagnet, the WLM and FLM volumes, and the coil current on the WLM shooting distance was studied. Furthermore, the capability of control over the WLM manipulation was confirmed by examining the WLM launch in different directions. Below, a summary of the results is provided:

- The collision of two liquid marbles is inelastic and consists of four stages. First, the FLM approaches the electromagnet with an accelerating motion while the WLM is stationary. Then, the driver FLM impulses the WLM and transfers a portion of its momentum to the driven liquid marble. Afterward, the stuck-together liquid marbles move toward the electromagnet with an overall accelerating motion and a velocity fluctuation. At last, the liquid marbles are detached: The FLM stops moving and is fixed on the coil tip, but the WLM exhibits a deaccelerating motion and finally stops due to the existence of friction force.
- By increasing the WLM initial distance from the electromagnet from 0 to 20 mm, its shooting distance increases by 50%, 70%, and 90% for liquid marbles volumes of 10, 30, and 50 μL, respectively. Additionally, when the WLM and FLM volumes increase, the WLM shooting distance increases as well.
- Increasing the FLM initial distance from the electromagnet results in an increment in the WLM shooting distance.
- As the WLM volume increases, its shooting distance decreases.
- By employing larger FLMs, the WLM can be manipulated to farther distances from the electromagnet.
- If the coil current increases, the WLM will be launched farther.
- By proper adjustment of the WLM off-center from the connecting line between the FLM center and the electromagnet tip, precise control over the WLM direction is attained. Accordingly, as the adjusted off-center increases, the WLM is launched less strongly, leading to a shorter WLM shooting distance and a larger WLM shooting path angle.

To sum up, this study investigates the potential of using a ferrofluid liquid marble as a manipulator for the actuation of nonmagnetic objects, particularly nonmagnetic liquid marbles. The current work also opens up opportunities for further research in integrating the manipulation, coalescence, and splitting of liquid marbles all in one platform. This is an interesting phenomenon with rich underlying physics that opens the door for future studies for a better understanding of the dynamics of the collision. Moreover, a 3D digital microfluidic manipulation can be developed for further investigations [60,61,62]; for instance, one can study the levitation of a WLM by using a few FLMs in different directions facing each other. Low friction, low evaporation rate, and capability of rolling even on hydrophilic surfaces have made liquid marbles ideal for employment in lab-on-chip platforms, which could be utilized for biomedical and agricultural purposes.

## Supplementary Materials

The following supporting information can be downloaded at https://www.mdpi.com/article/10.3390/mi14010049/s1. Video S1: The sideview of the launching process of a WLM by an FLM; Video S2: Nonstraight impact of a WLM with an FLM.
